# Differential Susceptibility of Germ and Leydig Cells to Cadmium-Mediated Toxicity: Impact on Testis Structure, Adiponectin Levels, and Steroidogenesis

**DOI:** 10.1155/2017/3405089

**Published:** 2017-12-20

**Authors:** Marli C. Cupertino, Rômulo D. Novaes, Eliziária C. Santos, Ana C. Neves, Edson Silva, Juraci A. Oliveira, Sérgio L. P. Matta

**Affiliations:** ^1^Department of General Biology, Federal University of Viçosa, 36570-000 Viçosa, MG, Brazil; ^2^Institute of Biomedical Sciences, Department of Structural Biology, Federal University of Alfenas, 37130-001 Alfenas, MG, Brazil; ^3^School of Medicine, Federal University of Jequitinhonha and Mucuri Valleys, 39100-000 Diamantina, MG, Brazil; ^4^Department of Basic and Health Sciences, Federal University of Jequitinhonha and Mucuri Valleys, 39100-000 Diamantina, MG, Brazil

## Abstract

This study investigated the relationship between germ and Leydig cell death, testosterone, and adiponectin levels in cadmium-mediated acute toxicity. Cadmium chloride was administered in a single dose to five groups of rats: G1 (0.9% NaCl) and G2 to G5 (0.67, 0.74, 0.86, and 1.1 mg Cd/kg). After 7 days, the animals were euthanized, and the testosterone and testes were analyzed. Dose-dependent Cd accumulation in the testes was identified. At 0.86 and 1.1 mg/kg, animals exhibited marked inflammatory infiltrate and disorganization of the seminiferous epithelium. While Leydig cells were morphologically resistant to Cd toxicity, massive germ cell death and DNA oxidation and fragmentation were observed. Although numerical density of Leydig cells was unchanged, testosterone levels were significantly impaired in animals exposed to 0.86 and 1.1 mg Cd/kg, occurring in parallel with the reduction in total adiponectins and the increase in high-molecular weight adiponectin levels. Our findings indicated that Leydig and germ cells exhibit differential microstructural resistance to Cd toxicity. While germ cells are a primary target of Cd-induced toxicity, Leydig cells remain resistant to death even when exposed to high doses of Cd. Despite morphological resistance, steroidogenesis was drastically impaired by Cd exposure, an event potentially related to the imbalance in adiponectin production.

## 1. Introduction

As life-threatening inorganic pollutants are widely distributed in soil, water, and food, heavy metal poisoning is a serious public health problem worldwide [1]. Due to the long half-life (20–40 years) and low rate of excretion by organisms, cadmium (Cd) is an environmental toxicant with great potential to induce irreversible injuries in multiple organs, especially the liver, kidney, brain, lungs, and testes [2–4].

In the testes, besides inducing microstructural fragility (e.g., endothelial damage, vascular congestion, edema, hemorrhage, and testis calcification) [5, 6], Cd also disturbs the redox balance, eliciting proinflammatory and prooxidant events linked to genotoxicity, carcinogenesis, and cell death [1, 2]. Cadmium-mediated toxicity has also been associated with inhibition of expression of important regulatory molecules, such as focal adhesion kinase, Src kinases, and tyrosine phosphatase SHP2; these are essential for maintaining the morphofunctional integrity of the germinative epithelium, especially the Sertoli cell barrier and Sertoli-germ cell interactions [7–10]. These changes are related to molecular imbalance of the testis microenvironment and have a negative impact on steroidogenesis and spermatogenesis; this results in severe consequences such as hypogonadal syndromes and disturbance of male fertility [11, 12].

There is consistent evidence relating oxidative stress and the inflammatory process to the microstructural and functional testis disturbances triggered by exposure to heavy metals [6, 13, 14]. Although poorly understood, adiponectins have been suggested to be immunomodulator molecules with important effects on testicular homeostasis [15–17]. Adiponectins are a class of hormones primarily produced by adipose tissue; their testis mRNA expression and protein levels have been associated with interstitial Leydig cells [15–17]. By interacting directly with Leydig cell receptors (Adipo-R1 and Adipo-R2), adiponectins act as potential endogenous regulators of steroidogenesis in animals and humans [17, 18]. As potent anti-inflammatory mediators, adiponectins also protect Leydig cells against cytokine-mediated cytotoxicity, acting as a testicular defense mechanism to attenuate the negative impact of proinflammatory molecules, particularly those released by macrophages (e.g., interleukin 1 [IL-1], tumor necrosis factor alpha [TNF-*α*], and interferon gamma [IFN-*γ*]), on steroidogenesis [19, 20]. To the best of our knowledge, the role of adiponectins in Cd-mediated toxicity of reproductive organs remains unexplored. Thus, investigation of the relationship between adiponectins, testis structure, and function can introduce a new perspective on the regulatory mechanisms activated in the testes in response to Cd toxicity.

Although the impact of Cd on the testis microstructure has been investigated [10, 21], the mechanisms related to the pathological reorganization of testis parenchyma and stroma remain poorly understood. Given that fertility disturbances have been reported after exposure to heavy metals, germ and Leydig cells have been considered the main testicular targets of Cd-mediated toxicity [12, 22]. However, it remains unclear if the heterogeneous distribution of germ and Leydig cells is also related to differential morphofunctional resistance or susceptibility to Cd toxicity. Thus, given the hypothesis that Cd acts as an endocrine disruptor [22, 23], this study investigated the relationship between testicular microstructural remodeling, cell death, steroidogenesis, and adiponectin levels in an experimental model of acute Cd-mediated toxicity.

## 2. Materials and Methods

### 2.1. Groups and Model of Cd-Induced Testicular Damage

Thirty male Wistar rats (9 weeks old) weighting 287.9 ± 14.14 g were maintained in animal facilities with controlled relative air humidity (60–70%), temperature (22 ± 2°C), and photoperiod (12/12 h). The animals received food and water ad libitum. The experimental protocol was approved by the Institutional Ethics Committee (protocol 030/2010).

The animals were randomized into five groups of six animals each: control (CT), 0.9% NaCl; Cd1, 0.67 mg Cd/kg (1.1 mg/kg CdCl_2_); Cd2, 0.74 mg Cd/kg (1.2 mg/kg CdCl_2_); Cd3, 0.86 mg Cd/kg (1.4 mg/kg CdCl_2_); and Cd4, 1.1 mg Cd/kg (1.8 mg/kg CdCl_2_). Cadmium chloride (CdCl_2_, Sigma-Aldrich, MO, USA) was dissolved in distilled water and administered in a single intraperitoneal injection. Cadmium doses were determined considering the minimal dose (1.2 mg/kg CdCl_2_) effective to induce testicular damage [24]. As low Cd doses are more realistic and consistent with environmental contaminations, we used one dose lower and two doses higher than minimally necessary to cause testicular effects [6].

### 2.2. Euthanasia and Organ Collection

Seven days after Cd exposure, the animals were weighed and euthanized under deep anesthesia (5 mg/kg xylazine and 45 mg/kg ketamine, i.p.). Blood samples (5 mL) were collected by cardiac puncture in glass test tubes, and testosterone plasma levels were quantified. The testes were removed and weighed. The right testis was used for the measurement of testicular Cd accumulation, genomic DNA extraction, analysis of oxidative damage, and adiponectin quantification. The left testis was immersed in histological fixative (1 M paraformaldehyde and 1 M glutaraldehyde in 0.1 M phosphate buffer, 1 : 1, *v*/*v*) for 24 h [6]. The tunica albuginea was excised by using a scalpel and surgical tweezers and weighed, and its weight was subtracted from the total testicle weight, providing the gonadal parenchyma weight [7]. Then, the testes were used for histopathological and stereological analyses in bright-field microscopy [6].

### 2.3. Testicular Cadmium Content

Testicular cadmium content was determined by atomic absorption spectrophotometry and by energy-dispersive X-ray spectroscopy (EDS) [6, 25]. Testis samples were weighed and dried at 70°C until a constant dry weight was achieved. Dried samples were digested (30 min) in Erlenmeyer flasks with 1.5 mL concentrated HNO_3_ and 0.5 mL HClO_4_ (70%) using a plate heater, where the temperature was gradually increased from 70°C to 90°C. After digestion, the samples were diluted in deionized water (25 mL) and filtered. Cadmium concentration in each sample was determined using an atomic absorption spectrophotometer (Varian 220FS SpectrAA, Palo Alto, California, USA).

Relative Cd dose-dependent testicular accumulation was investigated by EDS using a scanning electron microscope (Leo 1430VP, Carl Zeiss, Jena, Thuringia, Germany) with an attached X-ray detector system (Tracor TN5502, Middleton, Wisconsin, USA) [26]. Small pieces of the testis from each animal were dehydrated at 60°C and coated with a thin film of evaporated carbon (Quorum Q150 T, East Grinstead, West Sussex, England, UK). The EDS microanalysis was performed at ×1000 magnification, with an accelerating voltage of 20 kV and a working distance of 19 mm [6]. Cadmium distribution was normalized considering the mean distribution of reference minerals (Na, Ca, K, P, Mg, Fe, and S) [27, 28].

### 2.4. Histopathological and Stereological Analyses

After albuginea removal, fixed testis samples were dehydrated in ethanol and embedded in glycol methacrylate (Leica Microsystems, Wetzlar, Germany). Three-micrometer-thick semiserial sections were obtained in a rotary microtome. To avoid analyzing the same histologic area, one out of every 20 sections was collected and used. Testis slices were stained with 1% toluidine blue/sodium borate [6]. Stereological estimations were obtained from digital images captured at different magnifications using a light microscope (Olympus BX-60, Tokyo, Japan) equipped with a digital camera (Olympus QColor-3; Tokyo, Japan) [29]. Stereological principles [30] were applied to investigate the general testis microstructure. The volume densities (Vv) (%) of seminiferous tubules, intertubules, blood vessels, lymphatic vessels, and connective tissue were estimated in 10 histological fields per animal (200x magnification) using a point counting method and the equation Vv = Pp/Pt, where Pp is the number of test points hitting the structure of interest and Pt is the total points in the test system (Pt = 266).

### 2.5. Leydig Cell Histomorphometry and Stereology

Testicular number and volume distribution of Leydig cells (LC) were estimated from point counting, direct measures of nuclear volume, and the proportions between the nucleus and cytoplasm. The mean diameter of the LC nucleus was obtained by computational planimetry for 50 cells per animal. Only cells with evident nucleoli were used as the reference in all quantifications. The nuclear volume (LCnv) was obtained by calculating the mean nuclear diameter using the notation 4/3 *πR*^3^, where *R* = nuclear diameter/2. Using ×100 objective lens (×1000 magnification), volume densities of the LC cytoplasm (Vvcy) (%) and nuclei (Vvnu) (%) were estimated by point counting, using the same formula previously described. Leydig cell cytoplasmic volume (LCcyv) was estimated as follows: LCcyv = (Vvcy × LCnv)/Vvnu. These parameters were used to calculate the nucleus/cytoplasm ratio, which was estimated as LCnv/LCcyv. In addition, individual LC volume (LCV) was estimated as LCnv + LCcyv. Leydig cell volume was used to estimate the LC number per testis unity and testis mass. Leydig cell number per testis was estimated as LCV/gonadal volume, and LC number per mass unity was calculated as LCV/gonadal mass. All microstructural parameters were measured using the image analysis software Image-Pro Plus (Media Cybernetics, Rockville, Maryland, USA) [30].

### 2.6. In Situ Apoptosis Assay

Apoptosis was detected by the TUNEL (terminal deoxynucleotidyl transferase dUTP nick end labeling) assay (Calbiochem, Merck KGaA, Darmstadt, Germany). Testis samples were included in paraffin, and five-micrometer-thick sections were deparaffinized, rehydrated, and incubated with proteinase K for 20 min at room temperature. Sections were washed in distilled water and incubated with H_2_O_2_ plus methanol (5 min) to stop endogenous peroxidase activity. Preparations were incubated in a humid chamber with terminal transferase (Tdt) equilibrium buffer, at room temperature, for 20 min, followed by Tdt mix enzyme and dithiothreitol (DTT) for 60 min at 37°C. Apoptotic cells were detected from the formation of brown precipitate after incubation with 3,3-diaminobenzidine tetrahydrochloride (DAB) and H_2_O_2_ for 13 min. The terminal transferase enzyme was omitted for the negative control. Positive controls were incubated with 1.00 U/mL DNase I (Invitrogen, Waltham, Massachusetts, USA) in DNase buffer for 10 min. Tissue distribution of apoptotic cells was evaluated by computational analysis (Image-Pro Plus, Media Cybernetics, Rockville, Maryland, USA) determining the optical density of brown pixels in all images submitted to the TUNEL assay.

### 2.7. DNA Fragmentation Assay

Genomic DNA extraction was carried out according to [31]. Briefly, testis samples (100 mg) were macerated and treated with DNA extraction solution (100 mM Tris-HCl (pH 8.0), 50 mM EDTA, 500 mM NaCl, 30 *μ*L 20% sodium dodecyl sulfate (SDS), and 20 *μ*L proteinase K) at 65°C for 24 h. After inactivation in a dry bath at 95°C, 1.5 *μ*L 20 mg/mL of RNAse was added and incubated at 37°C for 30 min. Proteins and cellular debris were precipitated with 300 *μ*L 6 M NaCl at 4°C for 15 min. After centrifugation (25,000 ×g, 20 min), an equal volume of phenol : chloroform : isoamyl alcohol (25 : 24 : 1) was added and centrifuged, and the upper aqueous layer containing the nucleic acid was collected. Nucleic acid was precipitated with absolute ethanol and 10 M ammonium acetate solution (pH 5.2) at −20°C. Samples were washed with 70% ethanol, centrifuged at 3000 ×g for 15 min, dried, and then suspended in 50 *μ*L Milli-Q water. DNA damage was evaluated in agarose gel electrophoresis [27]. Briefly, genomic DNA quantity and purity were determined in a NanoDrop system (Thermo Fisher Scientific, NanoDrop Products, Waltham, Massachusetts, USA). Finally, 1000 ng/*μ*L DNA and molecular marker (1000 bp DNA ladder, New England BioLabs Inc., Ipswich, USA) was subjected to 1.5% agarose gel (containing 0.5 *μ*g/mL gel red) electrophoresis (Advance, Tokyo, Japan). DNA ladder formation was visualized under a UV transilluminator (Vilber Lourmat, Cedex, France). The optical density of the agarose gels was determined by computational analysis (Image-Pro Plus, Media Cybernetics, Rockville, Maryland, USA).

### 2.8. Serum Testosterone Quantification

Plasma testosterone concentrations were obtained by chemiluminescence using a commercial diagnostic biochemical kit (Access Testosterone, Beckman Coulter, Brea, California, USA) and the instructions provided by the manufacturer. The measurements were obtained on Access II equipment (Beckman Coulter, Brea, California, USA). The results were expressed in ng/dL.

### 2.9. Enzyme-Linked Immunosorbent Assay (ELISA) for Adiponectins

Fragments of testicular tissue (100 mg) were homogenized (LabGEN YO 0427-09, Cole-Parmer, Vernon Hills, IL, USA) in a protease inhibitor (Sigma-Aldrich, USA) and centrifuged at 3000 ×g for 10 min. The supernatant was collected for the cytokine assay. The concentration of cytokines was measured by the sandwich ELISA [32]. Adiponectin and high-molecular weight (HMW) adiponectin were measured using commercial kits, according to the manufacturer's instructions (USCN Life Science Inc., Wuhan, China).

### 2.10. Statistical Analysis

Results were expressed as means and standard deviations (SD). Biochemical and molecular data were submitted to unifactorial one-way analysis of variance followed by the Tukey post hoc test for multiple comparisons. Morphological data were compared using the Kruskal-Wallis test. All results with *p* < 0.05 were considered statistically significant.

## 3. Results

**Figure 1:**
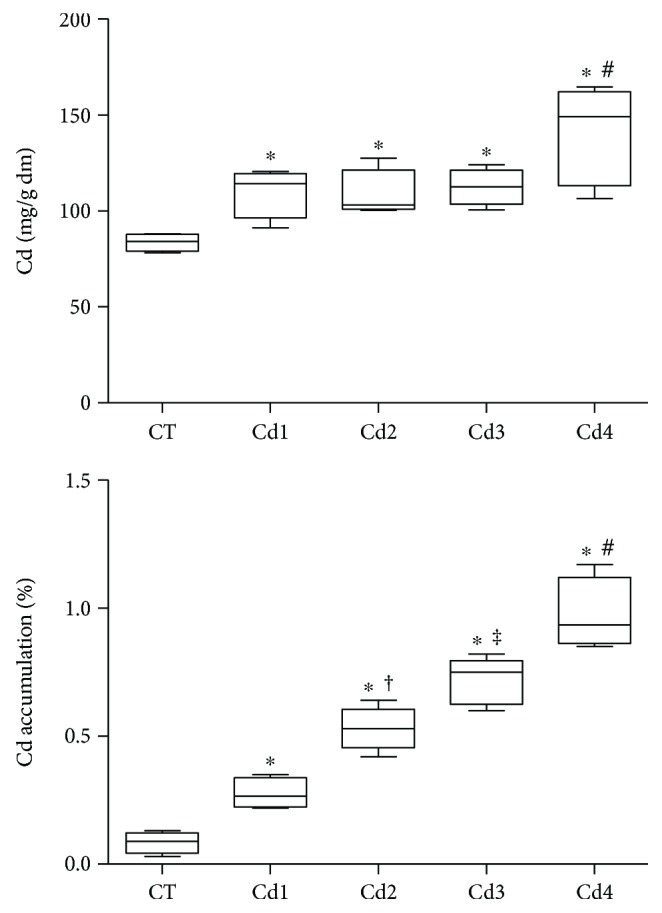
Cadmium (Cd) concentration (mg/g dry mass) and testicular dose-dependent accumulation (%) in rats. Cadmium accumulation was based on proportional distribution compared to the mean distribution of reference minerals (Na, Ca, K, P, Mg, S, Zn, Cu, and Se). Control (CT): 0.9% saline; Cd1: 0.67 mg Cd/kg; Cd2: 0.74 mg Cd/kg; Cd3: 0.86 mg Cd/kg; and Cd4: 1.1 mg Cd/kg. Statistical difference (^∗^*p* < 0.05 versus CT; ^†^*p* < 0.05 versus Cd1; ^‡^*p* < 0.05 versus CT, Cd1, and Cd2; and ^#^*p* < 0.05 versus CT, Cd1, Cd2, and Cd3).

The mean weight of the animals was similar (*p* > 0.05) in all groups (CT: 304.1 ± 21.2 g; Cd1: 300.2 ± 10.3 g; Cd2: 285.5 ± 40.0 g; Cd3: 279.7 ± 19.3 g; and Cd4: 270.2 ± 22.6 g). All groups treated with CdCl_2_ presented with increased testicular Cd concentration compared to CT animals (*p* < 0.05). The highest Cd concentration was identified in Cd4 animals, compared to all other groups (*p* < 0.05). After normalization by reference minerals, Cd accumulation showed a dose-dependent behavior, with the highest relative distribution of this metal in Cd4 animals (*p* < 0.05) (see Figure 1).

**Figure 2:**
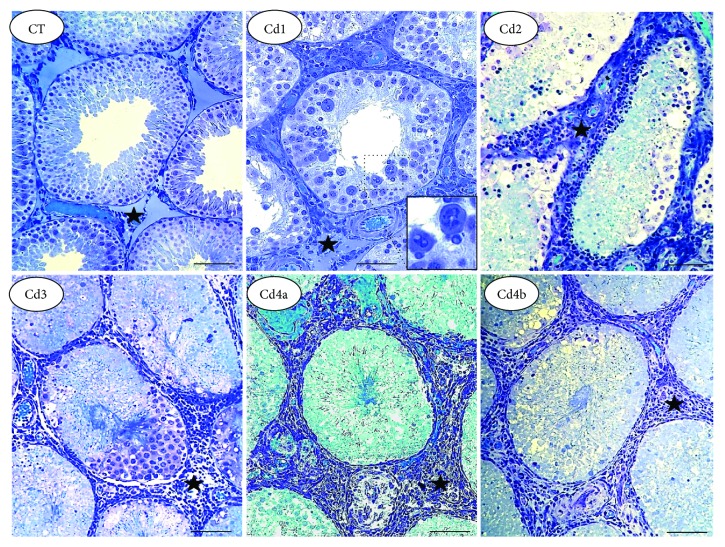
Representative microscopic images of the tubular compartment in the testis from control rats and those exposed to cadmium (Cd). Control (CT): 0.9% saline; Cd1: 0.67 mg Cd/kg; Cd2: 0.74 mg Cd/kg; Cd3: 0.86 mg Cd/kg; and Cd4: 1.1 mg Cd/kg. In CT, well-defined tubular structure with the preserved seminiferous epithelium is observed. In Cd1 to Cd4, dose-dependent epithelial damage is observed, with intense germ cell dissociation and reduced distribution. In Cd1, germ cells with abnormal nuclear morphology are highlighted. Marked inflammatory infiltrate is observed in intertubular compartment (star), especially in Cd2 to Cd4.

The testes from CT animals presented with well-delimited tubular and intertubular compartments (Figure 2). In these animals, regular seminiferous tubule profiles were observed and a thick and organized seminiferous epithelium, structured by well-defined and juxtaposed germ cells, was also noted. Animals treated with CdCl_2_ showed dose-dependent histopathological manifestations. The tubular compartment, nuclear hyperchromasia, cytoplasmic vacuolization, inflammatory infiltrate, epithelial dissociation, and germ cell fragmentation were the main histopathological findings observed in all intoxicated animals, especially in Cd3 and Cd4 (Figure 2).

The testes exhibited reduced mass in all Cd-exposed animals, compared to the CT group (*p* < 0.05). Albuginea mass was similar in all groups (*p* > 0.05). No statistically significant differences in tubular and intertubular stereological parameters were identified among the groups (*p* > 0.05) (see Table 1).

**Figure 3:**
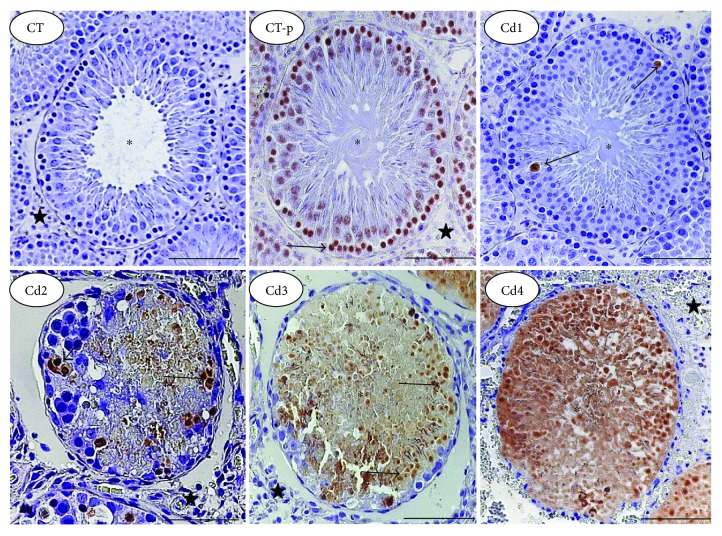
Distribution of apoptotic cells in the testes of control rats and those exposed to cadmium (Cd) (TUNEL technique, bars = 100 *μ*m). Control (CT): 0.9% saline; CT-p (positive control for the TUNEL technique): 1.00 U/mL DNase I; Cd1: 0.67 mg Cd/kg; Cd2: 0.74 mg Cd/kg; Cd3: 0.86 mg Cd/kg; and Cd4: 1.1 mg Cd/kg. Cells with brown nuclei show positivity for apoptosis (arrows). Asterisks: seminiferous tubules; star: intertubular compartment.

The TUNEL assay revealed germ cell death in all Cd-intoxicated groups (Figure 3). Positive cells were clearly identified from a well-defined brown marking, considering the negative and positive internal controls applied in this technique. Diffuse and massive distribution of TUNEL-positive germ cells was evident in all groups exposed to Cd, especially in Cd3 and Cd4. All intertubular cells were negative in the TUNEL method (Figure 3).

**Figure 4:**
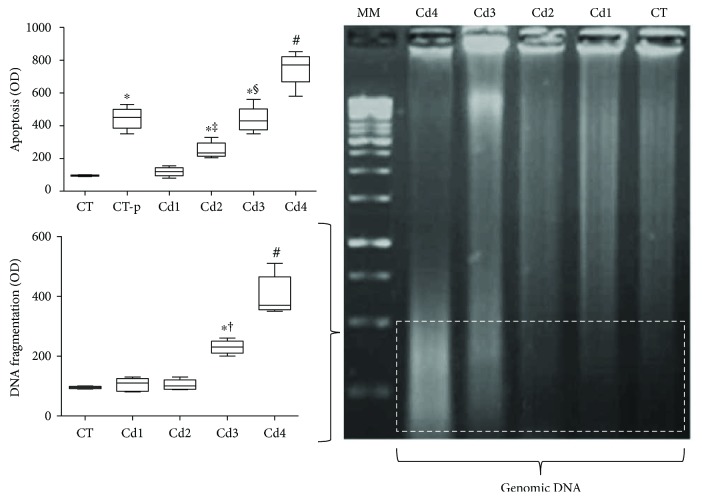
Genomic DNA oxidation and apoptotic index in the testis from control rats and those exposed to cadmium (Cd). DNA oxidation was evaluated by a ladder test using electrophoresis in agarose gel (left image). Optical density was computationally evaluated from the third tercile of agarose gel (dotted area). Apoptosis was evaluated by using microscopic images obtained from testis histological sections submitted to the TUNEL technique. In the graphics, DNA oxidation and apoptosis results were expressed as the difference in optical density (OD) compared to those of negative control animals (CT). MM: molecular marker. Control (CT): 0.9% saline; CT-p: 1.00 U/mL DNase I (positive control for the TUNEL technique); Cd1: 0.67 mg Cd/kg; Cd2: 0.74 mg Cd/kg; Cd3: 0.86 mg Cd/kg; and Cd4: 1.1 mg Cd/kg. In the graphics, data are expressed as mean and standard deviation (mean ± SD). Statistical difference (^∗^*p* < 0.05 versus CT; ^‡^*p* < 0.05 versus CT-p and Cd1; ^§^*p* < 0.05 versus CT-p, Cd1, and Cd2; ^#^*p* < 0.05 versus CT, CT-p, Cd1, Cd2, and Cd3; and ^†^*p* < 0.05 versus Cd1 and Cd2).

From computational analysis, dose-dependent cell death was clearly observed in all groups exposed to Cd, especially in Cd3 and Cd4 animals (*p* < 0.05), which also presented marked DNA fragmentation compared to the other groups (*p* < 0.05) (Figure 4).

**Figure 5:**
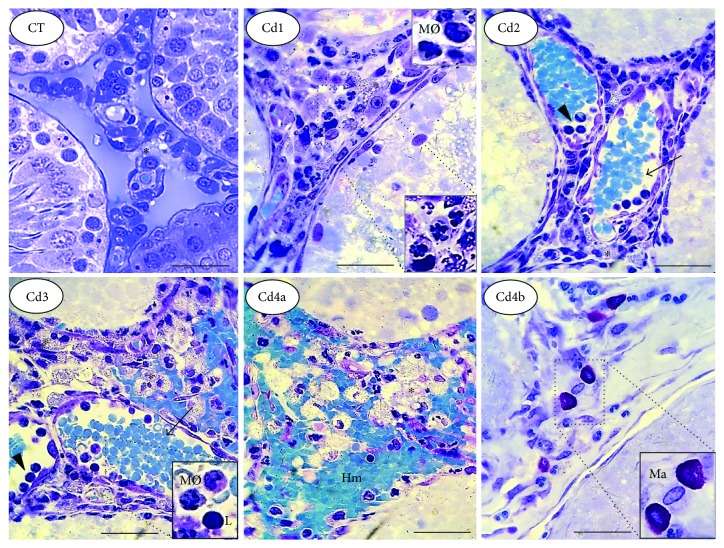
Representative microscopic images of the intertubular compartment in the testis from control rats and those exposed to cadmium (Cd). Control (CT): 0.9% saline; Cd1: 0.67 mg Cd/kg; Cd2: 0.74 mg Cd/kg; Cd3: 0.86 mg Cd/kg; and Cd4: 1.1 mg Cd/kg. In CT, well-defined intertubular structure with reduced cellularity and evident blood and lymphatic vessels is observed. In Cd1 to Cd4, dose-dependent damage is evident. In Cd-intoxicated animals, abnormal nuclear morphology in interstitial cells (Cd1, highlighted image), vascular congestion (Cd2 and Cd3), hemorrhage (Cd4a), and mast cell accumulation (Cd4b) were the main histopathological findings. The images of Cd4a and Cd4b indicate different and complementary morphological aspects of the same group of animals (Cd4). Arrows: blood vessels; arrowheads: leucocytes with marginal interaction; asterisk: Leydig cells; Hm: hemorrhagic foci; MØ: macrophages; L: lymphocytes; Ma: mast cells.

The testes from CT animals presented with well-delimited intertubular space, evident blood and lymphatic vessel profiles, homogeneous connective tissue distribution, reduced cellularity, and well-defined Leydig cells. Mononuclear and polymorphonuclear inflammatory infiltrate, vascular congestion, hemorrhage, and increased mast cell distribution were the main types of microstructural intertubular damage identified in Cd-intoxicated groups, especially in Cd3 and Cd4 (Figure 5).

Quantitative analysis indicated minor microstructural changes in Leydig cells. Only Cd3 and Cd4 animals presented with reduced nuclear diameter as well as reduced nuclear, cytoplasmic, and general cell volume, compared to the other groups (*p* < 0.05). The other microstructural variables presented similar results among the groups (*p* > 0.05) (see Table 2).

**Figure 6:**
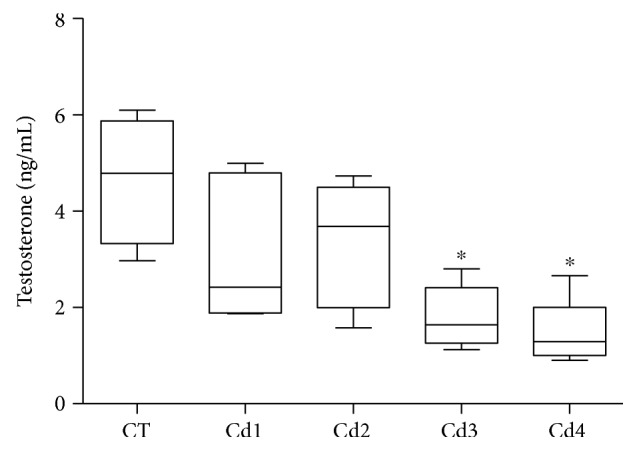
Testosterone serum levels in control rats and those exposed to cadmium (Cd). Control (CT): 0.9% saline; Cd1: 0.67 mg Cd/kg; Cd2: 0.74 mg Cd/kg; Cd3: 0.86 mg Cd/kg; and Cd4: 1.1 mg Cd/kg. Data are expressed as mean and standard deviation (mean ± SD). Statistical difference (^∗^*p* < 0.05 versus CT).

The groups CT, Cd1, and Cd2 presented with similar testosterone serum levels (*p* > 0.05). Testosterone was markedly reduced in Cd3 and Cd4 animals, compared to the other groups (*p* < 0.05) (see Figure 6).

**Figure 7:**
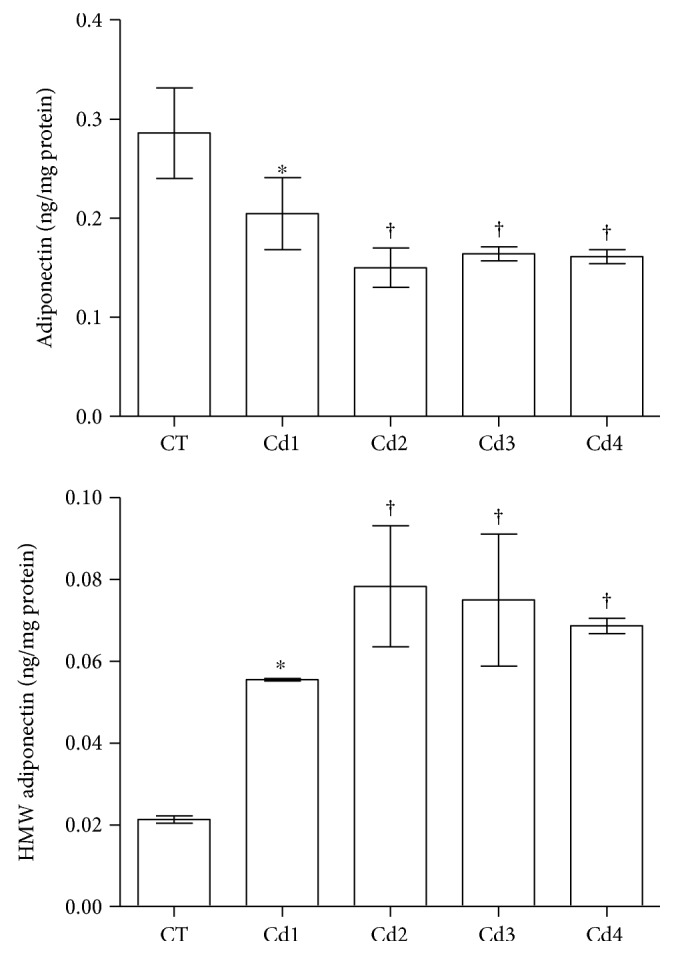
Testicular levels of adiponectin and high-molecular weight (HMW) adiponectin in control rats and those exposed to cadmium (Cd). Control (CT): 0.9% saline; Cd1: 0.67 mg Cd/kg; Cd2: 0.74 mg Cd/kg; Cd3: 0.86 mg Cd/kg; and Cd4: 1.1 mg Cd/kg. Data are expressed as mean and standard deviation (mean ± SD). Statistical difference (^∗^*p* < 0.05 versus CT; ^†^*p* < 0.05 versus CT and Cd1).

While total adiponectin testicular levels were reduced (*p* < 0.05), high-molecular weight adiponectin was increased in all Cd-intoxicated groups, compared to CT animals (*p* < 0.05). These findings were also replicated when comparing Cd2, Cd3, and Cd4 groups with Cd1 animals (*p* < 0.05) (see Figure 7).

## 4. Discussion

Taken together, our findings indicate that the experimental model of Cd-mediated toxicity was effective in inducing morphological, biochemical, and molecular abnormalities in the testes. Consistent with an acute model, marked testis damage was observed, particularly vascular congestion, intense inflammation, disorganization of the seminiferous epithelium, nuclear pleomorphism, cytoplasm vacuolization, and death of germ cells. Although reorganization of testis compartments was not achieved, the period of Cd exposure was enough to induce Cd accumulation and trigger genomic DNA fragmentation, antagonistic profiles of total adiponectin, and HMD adiponectin production, as well as disturbances in testosterone levels, which were associated with volumetric rather than numerical changes in Leydig cells.

As a typical model of heavy metal toxicity, Cd accumulation occurred in a dose-dependent manner [6]; this was consistent with morphological damage and molecular abnormalities in the testis (most severe in the groups Cd3 and Cd4). Although all groups exhibited testis hypotrophy, the proportions of tubular and intertubular components remained unchanged. This indicates that Cd induced similar volumetric effects on both testicular components, with no evident change in the relative distribution of testis structures. Conversely, acute Cd exposure was associated with marked histopathological damage, especially mononuclear and polymorphonuclear inflammatory infiltrate and microstructural abnormalities in germ cells. Inside the broad spectrum of heavy metal toxicity [33, 34], the testes seem to be highly sensitive to divalent metals, especially Cd [35]. There is evidence that increased susceptibility to Cd is associated with molecular mimicry and competition with Ca, which is essential for the maintenance of the integrity of the Sertoli cell barrier [4, 10]. As Cd and Ca exhibit divergent functional properties, the Sertoli cell barrier becomes unstable and unable to control the adluminal molecular environment; this causes germ cell stress, accumulation of free calcium, and cell death [6, 10]. De Souza Predes et al. [24] showed that a single dose of cadmium chloride (1 to 1.2 mg/kg) is sufficient to cause testicular degeneration, which was compatible with severe inflammation, diffuse hemorrhage, and germ cell vacuolization. Beyond these pathological manifestations, Bekheet [36] pointed out that disorganization of the seminiferous epithelium and germ cell death are the most severe pathological manifestations of Cd-mediated toxicity.

In fact, marked dose-dependent germ cell death was observed in animals exposed to Cd, which was more evident and diffused in the groups receiving the highest doses of Cd (Cd3 and Cd4). Surprisingly, TUNEL marking was restricted to the seminiferous epithelium, and no positive intertubular cells were identified. This finding indicates that heterogeneous distribution of parenchymal cells is associated with different resistance profiles against aggression and that germ cells are primary targets of Cd-mediated toxicity. As clearly identified, all differentiation stages of germ cells were susceptible to Cd-induced death. A similar characteristic was reported by Zhou et al. [37] and Marettová et al. [5] who found that beyond aberrant morphology, there were reduced spermatogonium and spermatocyte numbers, as well as abnormal and immature cells in the tubular lumen.

Although the signaling pathways underlying testis damage are not completely understood, cell death has been associated with direct and indirect Cd-mediated cytotoxicity [35, 38]. *In vitro* models indicate that in fibroblasts [39] and human leukemia cells [40], caspase activation is closely correlated with cell death, so that caspase inhibitors are effective in attenuating Cd-induced cell death. Lemarie et al. [41] and Shih et al. [42] showed that cells exposed to Cd exhibit increased gene expression and release of apoptosis-inducing factor (AIF), which triggers cell death. This mechanism was also observed in the testes from Cd-exposed rats, in which germ cell death was associated with nuclear membrane damage [43] and translocation of AIF from mitochondria to the nucleus [38]. By interacting with cell DNA, AIF induces chromatin condensation and fragmentation through recruitment of downstream caspase-independent nucleases, causing an electrophoretic pattern of ladder-like DNA splitting [38, 44]. In fact, our findings indicate that cell death was consistent with DNA fragmentation, especially in the groups Cd3 and Cd4. Beyond direct Cd-induced AIF-DNA interaction, genotoxicity has also been associated with Cd-mediated redox imbalance [43, 45]. Together with direct inhibition of antioxidant enzymes (e.g., superoxide dismutase, catalase, and glutathione-S-transferase), Cd also stimulates inflammation and the secondary production of reactive oxygen metabolites (ROS) from recruited leucocytes (e.g., respiratory burst), inducing intense lipid, protein, and DNA oxidation [6, 46]. Although cell death is closely correlated with redox status, steroidogenesis is also sensitive to oxidative stress, which is dependent on dose, duration, and frequency of heavy metal exposure [46, 47].

Beyond low testosterone levels, animals exposed to the highest doses of Cd (Cd3 and Cd4) also exhibited a reduction in absolute morphological parameters of Leydig cells. As this finding was not associated with Leydig cell death, the impact of these microstructural changes on steroidogenesis remains poorly understood. However, it has been reported that downregulation of steroidogenesis can precede morphological abnormalities, especially in the initial stages of Cd poisoning [35, 48, 49]. In fact, our findings indicate that, despite the marked microstructural resistance of Leydig cells, as compared to germ cells, steroidogenesis is potentially sensitive to Cd-mediated toxicity. This proposition is corroborated by Laskey and Phelps [50] who suggested that viability of Leydig cells is achieved at the expense of inhibition of metabolic pathways not directly involved in cell survival, including steroidogenesis. In addition, Cd was proposed as a direct endocrine disruptor, with negative impacts on testosterone production [23]. Although poorly understood, Cd-induced steroid biosynthesis imbalance is potentially associated with downregulation of steroidogenic acute regulatory proteins (StAR), which participate in the conversion of cholesterol to testosterone [51, 52]. It has been suggested that Cd also impairs the interaction between gonadotrophic luteinizing hormone (LH) and its receptor in Leydig cells [53]. However, this mechanism is controversial and requires further investigation.

Although the inhibitory effect of proinflammatory molecules, such as IL-1, IFN-*γ*, and TNF-*α*, on steroidogenesis is well known [15, 19], the testicular function of adiponectins is still poorly understood [17]. Even more obscure is the role adiponectins have in heavy metal poisoning. As a protective contraregulatory reaction against Cd-induced toxicity, we expected a consistent increase in adiponectin in the testes from animals exposed to the highest doses of Cd, especially Cd3 and Cd4. However, while total adiponectin was reduced, HMW adiponectin isoform was detected in high levels in all Cd-exposed animals. Although adiponectins present with potent immunomodulation and antiapoptotic and antioxidant properties [54, 55], our findings suggest that testicular adiponectins can have a role other than attenuation of inflammatory damage and germ cell death. Thus, a potential relationship between adiponectin and its HMW isoforms with StAR proteins brings further perspectives for the control of steroidogenesis and testis functionality [15, 17, 56]. By regulating StAR expression, Pfaehler et al. [56] suggested that adiponectin modulates steroidogenesis in Leydig cells by direct inhibition of gene transcription. In this study, downregulation of StAR was associated with reduced adiponectin levels (10 and 100 ng/mL) and low testosterone production in rats. In fact, adiponectin and its receptors have been identified in Leydig cells from animals and humans [15, 16]. Considering the variable profile of total and HMW adiponectins, it is possible that this molecule acts in different testicular pathways, an issue that requires further investigation. Conversely, there is evidence that testosterone selectively inhibits gene expression and secretion of HMW adiponectins [57], a finding potentially related to the high levels of these molecules in the groups receiving the higher doses of Cd, especially Cd3 and Cd4.

## 5. Conclusions

Taken together, our findings indicate that Cd-mediated toxicity is associated with morphological and functional testis damage. Beyond a heterogeneous distribution, germ and Leydig cells exhibited divergent profiles of resistance to Cd. While germ cells are highly susceptible and constitute a primary target of Cd-induced genotoxicity, Leydig cells are resistant to cell death. Although Leydig cells are resistant to Cd-induced cell death, even in high doses of this metal, steroidogenesis is profoundly impaired. Despite their anti-inflammatory potential, total and HMW adiponectins are potentially involved in testis function, including steroidogenesis, instead of exerting a protective role against Cd-mediated microstructural damage and germ cell death.

## Figures and Tables

**Table 1:** Biometric and stereological testicular parameters in control rats and those exposed to cadmium (Cd).

Parameters	CT	Cd1	Cd2	Cd3	Cd4
Testis (g)	1.53 ± 0.09	0.89 ± 0.22^∗^	0.83 ± 0.21^∗^	1.0 ± 0.11^∗^	0.94 ± 0.20^∗^
Albuginea (g)	0.07 ± 0.01	0.05 ± 0.01	0.07 ± 0.03	0.1 ± 0.05	0.1 ± 0.07
Tubule (%)	92.9 ± 0.17	88.9 ± 7.5	79.7 ± 11.5	88.5 ± 7.6	85.8 ± 8.4
Intertubule (%)	7.1 ± 0.2	11.1 ± 7.5	20.4 ± 11.5	11.5 ± 7.6	14.2 ± 8.4
Blood vessels (%)	4.6 ± 2.9	5.6 ± 4.9	4.5 ± 2.1	3.4 ± 2.2	2.9 ± 0.7
Lymphatic vessels (%)	46.4 ± 2.6	39.2 ± 20.0	28.5 ± 19.6	54.9 ± 6.9	33.4 ± 22.4
Connective tissue (%)	14.1 ± 5.2	16.2 ± 9.0	47.3 ± 30.0	30.7 ± 32.3	40.3 ± 35.9

Control (CT): 0.9% saline; Cd1: 0.67 mg Cd/kg; Cd2: 0.74 mg Cd/kg; Cd3: 0.86 mg Cd/kg; and Cd4: 1.1 mg Cd/kg. Data are expressed as mean and standard deviation (mean ± SD). Statistical difference (^∗^*p* < 0.05 versus CT).

**Table 2:** Leydig cell absolute microstructural parameters obtained from the testis of control and exposed rats to four different Cd concentrations.

Parameters	Control	Cd1	Cd2	Cd3	Cd4
Nuclear diameter (*μ*m)	6.4 ± 0.2	5.88 ± 0.3	5.6 ± 0.9	5.5 ± 0.3^∗^	5.2 ± 0.3^∗^
Nuclear volume (*μ*m^3^)	146.2 ± 20.6	121.1 ± 19.4	113.2 ± 26.2	108.3 ± 10.7	101.1 ± 9.1^∗^
Cytoplasm volume (*μ*m^3^)	500.1 ± 105.1	394.6 ± 135.9	306.4 ± 108.1	268.2 ± 98.6^∗^	201.0 ± 150.3^∗^
Cell volume (*μ*m^3^)	646.3 ± 103.5	515.7 ± 149.9	419.6 ± 132.3	376.5 ± 105.4^∗^	302.1 ± 152.4^∗^
Cells per testis (×10^8^)	7.6 ± 2.2	10.8 ± 5.9	6.1 ± 3.6	8.4 ± 4.4	12.2 ± 4.7
Cells per testis gram (×10^8^)	6.1 ± 3.0	11.9 ± 6.7	7.0 ± 3.2	7.3 ± 3.5	13.4 ± 5.9
Nucleus/cytoplasm ratio	21.4 ± 1.2	23.98 ± 5.3	28.5 ± 10.8	28.4 ± 11.3	31.7 ± 12.4

Control (CT): 0.9% saline; Cd1: 0.67 mg Cd/kg; Cd2: 0.74 mg Cd/kg; Cd3: 0.86 mg Cd/kg; and Cd4: 1.1 mg Cd/kg. Data are expressed as mean and standard deviation (mean ± SD). Statistical difference (^∗^*p* < 0.05 versus CT).
